# Implementation of the Trigger Checklist and assertive outreach to reduce drug-related harm in the Emergency Department: A realist evaluation

**DOI:** 10.1371/journal.pone.0358880

**Published:** 2026-10-05

**Authors:** Michelle Beattie, Lesley Campbell, Bev Fraser, Anna Terje

**Affiliations:** 1 Centre for Rural Health Sciences, University of the Highlands and Islands, Inverness, Highlands, Scotland; 2 Drug and Alcohol Recovery Service, NHS Highland, Caithness, Highlands, Scotland; 3 Drug and Alcohol Recovery Service, NHS Highland, Inverness, Highlands, Scotland; University of Cambridge, UNITED KINGDOM OF GREAT BRITAIN AND NORTHERN IRELAND

## Abstract

**Background:**

Drug-related harm is a major global public health concern, with opioids implicated in over 70% of drug-related deaths. Scotland reports the highest drug-related mortality rate in Europe, with remote and rural communities facing additional barriers to accessing harm reduction and treatment services. Assertive outreach is increasingly recognised as a key strategy to engage individuals at risk who are disconnected from traditional services. This study evaluates the use of a Trigger Checklist to identify and outreach individuals within 48 hours of referral in a rural Emergency Department (ED).

**Methods:**

A realist evaluation explored how and why the Trigger Checklist works within the complexity of an ED setting. Guided by Pawson and Tilley’s framework, the evaluation followed three stages: theory gleaning, refining, and consolidation. Initial programme theories were developed using literature and advisory group input, then refined through 13 realist interviews with ED clinicians and outreach workers (OW). Data were analysed using Context-Mechanism-Outcome (CMO) configurations and linked to middle-range theories.

**Results:**

Three programme theories emerged: (1) Confident decision-making, where certainty, conversational aids, ethical reasoning, and outreach worker prompts influenced checklist use; (2) Trusting relationships, shaped by OWs’ approachability, situational awareness, and mutual respect; and (3) Perceived value, driven by altruism, narrative feedback, and perceived reductions in ED visits. Relational coordination theory explained how shared goals, knowledge, and respect – reinforced by timely, accurate communication – enabled coordinated action. OW presence and recovery discussions were critical to foster confident and appropriate referrals among ED clinicians.

**Conclusions:**

The Trigger Checklist can support timely outreach potentially reducing drug-related harm in rural ED settings, but its success depends on relational dynamics, perceived value, and organisational integration. To avoid checklist fatigue or tokenistic use, it should be embedded with the support of OWs who foster trust. These relationships will enhance use of the checklist as a meaningful intervention within complex care systems.

## Introduction

Drug use is estimated to contribute to more than half a million deaths globally each year, with opioids implicated in almost 80% of these fatalities [1]. The incidence of fatal opioid overdoses is rising internationally [2]. Scotland currently reports the 19.1 drug-related deaths for every 100,000 people in Scotland. While this is a 13% decline compared with 2023, the rate was 3.6 times as high in 2024 than 2000 [3]. Despite inconsistencies in global data recording practices, Scotland continues to have a higher rate of drug-related deaths per million population than most countries internationally [4]. The harms associated with drug use extend beyond the individual, affecting families, communities, and placing significant strain on health and social care systems. Those living in remote and rural areas may face additional challenges arising from geographical barriers to service access, difficulties in recruiting specialist drug-recovery practitioners, and limitations in local digital infrastructure [5].

Reducing drug-related harm necessitates a multifaceted approach encompassing prevention, early intervention, harm reduction, and emergency response. Across Europe, harm reduction strategies have been widely implemented, including access to opioid agonist treatment (OAT), the distribution of naloxone for overdose reversal, provision of sterile injecting equipment, and the establishment of supervised consumption facilities, [4]. Despite variations in national approaches, there is a broad consensus within health policy frameworks that medication-assisted treatment (MAT) represents the most effective response to opioid dependence.

In the United States, recent legislative reforms have aimed to expand access to MAT by removing regulatory barriers and promoting provider education [6]. Canada’s national strategy similarly supports proactive identification of individuals at risk and the provision of MAT within a public health framework [7]. In the United Kingdom, the “All Our Health” [8,9] framework emphasises that every contact with health services is an opportunity to engage individuals in treatment, while Scotland has introduced national MAT Standards to improve consistency and access. Notably, Standard 3 states that “all people at high risk of drug-related harm are proactively identified and offered support to commence or continue MAT” [9].

Despite alignment across National and international policy, the practical challenge of identifying and engaging individuals at risk of drug-related harm in treatment remains significant. While there is recognition that the circumstances experienced by people who use drugs can be characterised by significant instability, which can impede consistent engagement with services [10,11], there is also growing awareness that structural barriers within health and social care systems contribute to this disconnect. These challenges are likely exacerbated in remote and rural communities. Fragmented organisational infrastructures, siloed service delivery, and limited interagency collaboration reduce opportunities for timely intervention. Addressing these issues is essential to improving access, retention, and outcomes in medication-assisted treatment and broader harm reduction efforts.

Assertive outreach is increasingly recognised as a critical strategy in reducing drug-related harm, particularly among people who are disengaged from traditional services and experience marginalisation. Individuals who use drugs often face systemic barriers – including stigma, criminal behaviour, disconnect from social supports and fragmented service provision – which diminishes access to statutory support [12,13]. The stigma associated with drug use is often amplified in rural communities, where close-knit social networks and limited anonymity can intensify the visibility of individuals who use drugs, exacerbating exclusion and reinforcing barriers to access support. A recent study found that individuals who use drugs and reside in remote or rural areas are at increased risk of experiencing non-fatal overdoses [14].

Proactive outreach aims to bridge the gap by identifying individuals at risk and connecting them with appropriate interventions in a timely manner. A programme in Boston successfully outreached people by situating their mobile recovery service in an area with high overdose rates [15]. Peer-led outreach models have also shown promise; peer support workers conceptualise harm reduction as both a life-saving intervention and a pathway to recovery, offering emotional and practical support grounded in lived experience [16,17]. These approaches emphasise the importance of low-threshold and culturally responsive outreach services that meet people where they are, both geographically and in their recovery journey.

A need to develop a process to outreach those at risk of drug-related harm in a timely manner in a remote and rural area of Scotland was identified by the high incidence of drug-related harm. The Caithness Drug and Alcohol Recovery Service undertook some improvement work in 2023 to create, test and implement a Trigger Checklist to identify and outreach people within 48 hours of referral from police custody, the Emergency Department (ED) and other community partners.

The Trigger Checklist includes two screening questions to determine harmful drug use, ten risk assessment questions, and a free text section for additional comments from the referrer. The tool uses a low threshold for determining risk of drug harm with assertive outreach initiated for a ‘yes’ response to either of the two screening questions and to any one of the 10 risk questions. Details of the initial testing and development are reported elsewhere [18]. During initial testing and development very few Trigger Checklists were received from the ED despite anecdotal evidence of missed opportunities. It is known that many people at risk of drug harm are frequent attenders in the ED due to an increase in their ill health and accidents associated with their high-risk behaviour [19]. This study aimed to identify the reasoning of ED clinicians in response to the Trigger Checklist that might explain why, how and for whom the checklist could optimise outreach for those at risk of drug-related harm in a remote and rural ED.

## Methods

### Evaluation design

Realist evaluation is well-suited to exploring how and why the Trigger Checklist works within the complexity of an Emergency Department. Grounded in the work of Pawson and Tilley [20], this approach assumes that reality operates across three layers: the empirical (what is observed), the actual (what happens), and the real (underlying mechanisms). This emphasis on ontological depth is particularly relevant as it enables exploration beyond surface-level metrics and investigates the unseen phenomena (for example staff reasoning, organisational culture, and contextual triggers) that shape implementation and effectiveness. By doing so, it offers a nuanced understanding of how the checklist activates change within a complex system, and why it may succeed in some contexts but not others. Realist Context-Mechanism-Outcome (CMO) configurations were used as a heuristic to make sense of causal explanations underpinning Trigger Checklist completion within the ED.

Developing a realist evaluation framework begins with articulating the programme theory—that is, specifying how an intervention is expected to generate its effects and the conditions under which these effects are likely to occur. Programme theories are typically informed by prior research, practitioner experience, and the assumptions held by intervention designers about the mechanisms through which change will unfold. A clear and explicit understanding of an intervention’s intended aims, processes, and outcomes is therefore essential from the outset [21]. Within a realist perspective, whether an intervention ‘works’ depends on the decisions and actions taken by the individuals involved. These responses are shaped by the resources, opportunities, or constraints introduced by the intervention itself [20].

The evaluation followed the realist stages of theory gleaning, theory refining, and theory consolidation [22]. Ethical approval was sought and obtained from the University of the Highlands and Islands (ID: ETH2425−0561). Initial theory gleaning drew on literature informed by one author’s previous master’s dissertation (LC) and tacit knowledge from members of the Project Advisory Group (see PAG membership in Box 1). This informed the development of initial programme theories (IPTs) using CMO configurations (S1 File. Initial CMOs to final CMOs). Theory refining was iterative and interactive, involving interviews with ED staff and several data meetings with the research team. Theory consolidation involved retroduction, the process of inferring the underlying mechanisms that must be operating, given the observed outcomes in a particular context, and linking revised programme theories and CMOs to middle-range theories, the context specific explanations that link mechanisms to observed outcomes.

**Box 1 PAG Membership**

- Recovery Service Lead
- Senior Charge Nurse ED
- Person in Recovery
- ED Consultant
- Psychiatrist/ Lead for Recovery
- Realist Expert (out with project team).
- Research Project Lead
- Post Doc Researcher

### Service and setting

The rural community involved in this project has an adult population of 21,291, with the estimated prevalence of drug use in Scotland at 1.62% [23]. On average, 173 individuals are referred annually to the local recovery service, with approximately half of these referrals related to drug use. Applying the national prevalence rate to this community suggests that around 345 adults may be using drugs. This indicates that roughly three out of four people who use drugs in this area are not accessing treatment services. The nearest acute hospital is located over 100 miles away.

Drug use is closely associated with deprivation [24], and this community contains areas of significant deprivation. The locality has the highest rate of drug-related hospital admissions per capita within the Highland Health board area [25], further supporting the assumption that the recovery service is not currently reaching most people who use drugs. The Drug and Alcohol Recovery Service provide care for adults in the geographical area. The service is made up of a professional/ service lead nurse, three registered nurses and four outreach workers covering two separate towns. The service lead is a nurse prescriber and addictions consultant support is provided virtually.

### Participants

There is no strict ‘rule of thumb’ for the number of realist interviews to be conducted as interview data does not need to reach ‘data saturation’ but rather achieve relevance and rigor [22]. Participants were purposively sampled to ensure sufficient breadth of experience of the Trigger Checklist to ensure they could refute, refine and create programme theories (See Box 2 for Participant Criteria and Final Sample). The aim was to recruit approximately 10 stakeholders for individual realist interviews, with 13 ultimately participating.

**Box 2 Participant Criteria Sample**

**Table:** 

Outreach Worker	2
Paramedic	1
ED Senior Charge Nurse	1
ED Advanced Nurse Practitioner	1
ED Nurse	4
ED Doctor	2
ED Healthcare Support Worker	2
	**n = 13**

#### Recruitment

Recruitment was facilitated through the Clinical Nurse Manager and the Drug and Alcohol Recovery Service Team Lead. They shared information about the study during relevant Emergency Department (ED) and Drug and Alcohol Recovery meetings, as well as at informal training sessions. Individuals who expressed an interest were sent a Participant Information Sheet via email with details to contact either the Research Assistant (AT) or the Project Lead (MB) to arrange an interview.

#### Data collection

Interviews were offered in person within the ED on specific dates, or remotely via Microsoft Teams or telephone at a mutually agreed date/time between 21/03/2025 to the 05/05/2025. Of the 13 interviews conducted, four were via Microsoft Teams, one via telephone, and eight were conducted in person. Written informed consent was obtained from all participants prior to data collection. All interviews were audio-recorded and professionally transcribed verbatim.

Interviews were guided by a semi-structured topic guide (S2 File) and facilitated using the teacher–learner approach to realist interviewing [22]. Using this approach, the interviewer introduced theories and assumptions about how the Trigger Checklist was thought to work (acting as the “teacher”), while the interviewee shared their perceptions and experiences of how the checklist operates in practice (acting as the “teacher” in turn). This cyclical exchange continued until all relevant IPTs were explored and no new theories emerged.

#### Data analysis

Initial Programme Theories (IPTs), developed from the literature and the PAG, were iteratively refined through ongoing dialogue between the research team and further engagement with the literature (theory refining). This process continued until consensus was reached on the most relevant IPTs—particularly those suitable for exploration through interviews with clinicians from the Emergency Department (ED) and outreach workers.

Data were coded in NVivo 14 (MB, AT) around IPT groupings, e.g., those that related to patient presentation were coded under ‘patient presentation.’ Transcript data were then used to further develop the codes, and annotations were made for each IPT around explanatory mechanisms, specifically evidence of reasoning or actions that affirmed, refuted or refined the IPTs. Within the mechanism codes, contexts and outcomes were highlighted to observe patterns in the data.

Given the interpretative nature of realist evaluation, reflexive practice was embedded throughout data analysis. The research team, comprising of academic and clinical members, met weekly during theory refinement and consolidation to discuss emerging findings and CMO development. Particular attention was given to retroductive reasoning, with discussion focused on exploring alternative explanations and identifying underlying mechanisms that could plausibly account for the observed outcomes in specific contexts. These discussions supported multiple perspectives and final consensus building.

Theory consolidation involved two researchers independently reviewing the final theories. Memos were written to document retroductive ideas, decisions, and integration of middle-range theories to help make sense of the data.

## Results

The analysis identified three programme theories, each comprising of interrelated sub-themes. Rather than presenting these as linear or compartmentalised categories, the conceptual framework (S1 Fig) visually illustrates their interconnectedness, highlighting the dynamic relationships across contexts, mechanisms, and outcomes and the programme theories themselves.

### Relational co-ordination

Relational coordination was identified as a helpful middle-range theory that underpinned the findings and supports the transferability of the findings to similar contexts. The theory posits that relationships are a vital conduit for effective coordination, facilitated through shared goals, shared knowledge and mutual respect [26]. While shared knowledge is common in healthcare settings, it is insufficient on its own to build and sustain relational coordination. The communication dimensions of frequency, timeliness, accuracy and problem solving mutually reinforce each other and are essential for maintaining effective relationships. For example, the frequency of OW visits to the ED enabled timely opportunities for knowledge exchange and problem solving, which in turn fostered trust between ED clinicians and OWs. When the aspects of shared goals, shared knowledge and respect are present, they provide a foundation for collective identity and coordinated action [26].

**Confident decision-making** – including certainty, an aid to conversation, balancing risk and confidentiality and regular human prompts.
**Trusting relationships** – characterised by approachable outreach workers (OW), their situational awareness, and the development of trust and mutual respect between OW and ED clinicians.
3. **Perceived value** – encompassing altruism, the value of narrative feedback, and the perception of reduced ED visits.

The following paragraphs present and explain each programme theory, along with the CMO configurations (S1 Table) and detail of alterations from initial CMOs to the final CMOs (S1 File).

Programme theory 1 – Confident decision-making: ED staff feel confident in their decision-making to use the Trigger Checklist when it is relevant to the patient presentation, is used as an aid to a patient conversation around drug-related harm and OW are present twice daily in the Emergency Department.

Programme theory one was developed from seven CMOs: certainty (CMOs 1–3), an aid to conversation (CMO 4), confidentiality breach versus harm (CMOs 5&6), and regular human prompt (CMO 7).

### Certainty

The first sub-theme of confident decision-making highlights the importance of ED staff having certainty in identifying people who use drugs and in the appropriateness of referral, which influences whether they complete the Trigger Checklist. Data configurations revealed that ED staff were more likely to act when they recognised an increase in a person’s drug-related presentations—either due to concern for their welfare or familiarity from repeated attendances (CMO2, CMO3). This recognition signalled escalating drug-related harm and reinforced the appropriateness of referral decisions. Conversely, when patients presented with acute, potentially life-threatening conditions, staff often prioritised immediate clinical care, resulting in the Trigger Checklist not being completed (CMO1). An initial rival theory proposed that the severity of such conditions might prompt staff to complete the checklist more readily, as the urgency could act as a trigger for action. However, this initial theory was removed due to a lack of supporting data. This pattern suggests that personal familiarity and perceived escalation in risk of harm triggered emotional and professional responsibility, enhancing certainty in decision-making and prompting action to complete the TC.

### Aid to conversation

Initial programme theories suggested that the number of questions within the TC could possibly be reduced due to the administrative burden on the ED clinicians (see CMOs deleted in S1 File: Initial CMOs to final CMOs). However, the ten risk-related questions remained important to the Trigger Checklist use. Staff adapted its use, treating it as a conversational guide rather than a rigid script. Many found that asking all questions directly could hinder rapport, so they used the checklist flexibly—sometimes deferring parts of the conversation or reframing questions to suit the situation (CMO4). Over time, staff recognised that the checklist enhanced their awareness of drug-related risks and supported more sensitive, patient-led discussions. Even when not fully completed, the checklist supported proactive engagement and informed decision-making by scaffolding sensitive, patient-led discussions around drug harm.

### Confidentiality breach versus harm

The Trigger Checklist (TC) may be completed without a patient’s consent when a clinician determines that the level of vulnerability and risk justifies overriding the need for consent. This decision is guided by the Caldicott Guardian principles, particularly Principle 7, which states that the duty to share information for individual care is as important as the duty to protect [27].

Clinicians who were confident in applying these principles felt ethically justified in completing the TC without consent when concerned about significant drug-related harm (CMO5). For them, the decision was straightforward: preventing harm took precedence over maintaining confidentiality, enabling informed and ethically sound action. In contrast, clinicians less familiar with the Caldicott principles were more hesitant. Concerns about breaching confidentiality, damaging the therapeutic relationship, or facing litigation (CMO6) led some to avoid completing the TC altogether.

An initial programme theory suggested that including a statement on the TC reinforcing Caldicott Principle 7 might reduce anxiety around potential legal consequences. However, the data did not support this. Instead, the key mechanism was clinicians’ underlying beliefs – particularly their fear of litigation and their confidence in being able to justify their decisions. This suggests that knowledge of ethical and legal frameworks is not simply applied rationally; rather decisions are influenced by deeply held professional beliefs and historic education around fears of litigation.

### Regular human prompt

Outreach workers (OWs) acted as timely, human prompts for busy ED staff, encouraging consideration of potential referrals and facilitating conversations, especially when clinicians were uncertain about whether to complete a Trigger Checklist (TC) (CMO7). These interactions often helped shift clinicians from hesitation to confidence, especially when discussing the likelihood or risk of drug-related harm. Engaging with a OW enabled staff to clarify risk, resolve doubts, and feel more assured in their decision as to the appropriateness of a TC completion.

Initially, the role of OWs was understood to be limited to visiting the ED twice daily to collect completed TCs. As a result, no initial context-mechanism-outcome (CMO) configurations were developed around their involvement. However, patterns in the data revealed their pivotal role in influencing clinician decision-making, leading to the development of this CMO.

Programme theory one illustrates how ED staff decision making in using the Trigger Checklist is shaped by their certainty in identifying drug-related harm, ethical clarity around confidentiality, and knowledge and support from outreach workers. The checklist functions not only as a referral tool but also as a flexible aid for sensitive conversations, with its use influenced by both clinical judgement and relational dynamics within the ED. Programme theory 2 further explains the important relationship between OW and ED clinicians and the impact for people who use drugs that attend the ED.

Programme theory 2 – Trusting relationship: When the OWs have high situational awareness, they can judge the right moment and approach to getting the Trigger Checklist completed by ED staff. A mutually respecting relationship between the ED staff and OWs enables knowledge sharing which ultimately benefits the person who takes drugs.

Programme theory two was developed from five CMOs: approachable OWs (CMO 8), situational awareness of the OW (CMO 9), trust (CMOs 10&11), and mutual respect (CMO 12).

### Approachable outreach workers (OWs)

Patterns in the data revealed that the outreach workers (OWs) were consistently perceived as friendly, approachable, and supportive, which encouraged ED staff to engage with them about potentially drug-related patient presentations. Over time, these interactions fostered trusting relationships, enabling open and honest discussions about patients at risk of drug-related harm (CMO 8). ED staff also observed the compassionate and non-judgemental way OWs interacted with people who use drugs, which positively influenced their own perceptions and attitudes towards this group of people. Ultimately, the OWs’ approachability facilitated case conversations and supported more informed, person-centred decisions that led to the Trigger Checklist being (appropriately) completed.

### Situational awareness of the OW

outreach workers (OWs) demonstrated strong situational awareness, enabling them to judge the right moments to engage ED staff about potential referrals (CMO9). Rather than interrupting busy clinicians they used their own judgement and observations, for example reviewing the ED whiteboard to identify and note names for referral for later discussion. They also supported ED staff by helping complete the Trigger Checklist when needed. Importantly, the OWs’ understanding of the ED environment helped build strong relationships with ED staff—relationships that were essential to ensuring the Trigger Checklist was completed. Their situational awareness, timing, and collaborative approach fostered trust, enabling more timely and person-centred decisions around drug-related harm. These CMOs emerged from the data, as the initial programme theories did not anticipate the OWs’ active role. At the outset, the intervention was perceived as limited to the Trigger Checklist itself, with OWs simply collecting completed forms. The data revealed their influence to be far more significant.

### Trust

In this rural emergency department building a trusting relationship between staff and individuals presenting with drug-related issues is crucial for eliciting honest disclosures. Individuals may withhold information due to fear of judgment, shame, or concerns that their responses could lead to unwanted consequences such as family involvement, police action, or social services intervention—especially when there is a general mistrust of those who work in public services (CMO 10). However, when an outreach worker (OW) is present or when ED staff take time to build rapport, individuals begin to feel safer and more confident, which fosters trust and encourages more open and honest communication. The initial CMO identified the mechanism of fear but was revised to incorporate the mitigation enacted in the presence of the OW.

Similarly, in close-knit rural communities, trust between ED staff and OSWs takes time to develop but is essential for legitimising the sharing of sensitive information. This mutual trust ensures that disclosures are made with the understanding that information will be handled respectfully and remain within the bounds of care services, which increases the completion of Trigger Checklists, ultimately improving outcomes for those at risk of drug-related harm (CMO 11). Again, the importance of a trusting relationship between the OW and ED staff only became apparent following data collection, hence a new CMO.

### Mutual respect

Interview data revealed a strong pattern of mutual respect between emergency department (ED) staff and outreach workers (OWs), with each valuing the other’s expertise in supporting people who use drugs. This mutual respect fosters a collaborative environment for shared knowledge and each professional’s contribution is recognised as essential to holistic care. When ED staff and outreach workers appreciate and respect each other’s distinct roles, it legitimises their shared efforts and strengthens trust, enabling sensitive information to be communicated appropriately. This respectful partnership ensures that patients receive timely physical care in the ED and are seamlessly connected to follow-up support through outreach services like DARS, ultimately improving outcomes for those at risk of drug-related harm (CMO 12).

Programme theory two unearths how trusting relationships between outreach workers (OWs) and Emergency Department (ED) staff enable effective use of the Trigger Checklist (TC) for patients at risk of drug-related harm. Developed from five CMOs, it highlights how OWs’ approachability, situational awareness, and mutual respect foster collaboration. OWs were consistently seen as friendly and non-judgemental, encouraging ED staff to engage in open discussions. Their ability to judge the right moment to approach staff, often by observing the ED environment, built trust and supported timely TC completion. In rural settings, trust between staff and patients was also critical, as fear and stigma often hindered disclosure. OWs helped mitigate these barriers, creating safer spaces for honest communication. Initially, the intervention was seen as checklist-only, but data revealed OWs played a pivotal role in ED staff completing the Trigger Checklist.

Programme theory 3 – Perceived value: ED staff are more inclined to complete the Trigger Checklist when they feel an intrinsic motivation and desire to support individuals at risk of drug-related harm beyond what standard emergency care provides. Their motivation increases when they perceive the checklist as a valuable tool- especially when they observe fewer attendances and receive narrative feedback from OWs about individuals who have been successfully supported.

### Altruism (CMO 13 and 14)

In this rural emergency department, altruism played a pivotal role in motivating staff to complete the Trigger Checklist for individuals presenting with potentially drug-related issues. When ED staff have experienced serious outcomes—such as a death or non-fatal overdose—the emotional impact deepens their empathy and reinforces the importance of early intervention. These experiences serve as powerful reminders of the value of proactive outreach in preventing future harm and prompt them to complete the Trigger Checklist (CMO 13). This was identified as an initial CMO but revised to incorporate the mechanism of value, as ED staff were clear that they were completing the checklist as they perceived it to be valuable.

Additionally, there was widespread recognition that people at risk of drug-related harm usually required additional interventions not provided by an ED. Clinicians were often limited in what they could offer so when ED staff had a clear and accessible referral process, completing the checklist became a meaningful act of care in a setting where long-term interventions are often limited. The combination of empathy and a desire to support vulnerable individuals drove staff to engage with the Trigger Checklist (CMO 14) not just as a procedural task, but as a compassionate response to reduce the trauma of drug-related harm for the individual, the clinician and the wider community.

### Value of narrative feedback (CMO 15 & 16)

Initial programme theories proposed that visually presenting quantitative process and outcome data in the ED (possibly as run charts) would motivate staff to complete the TC. However, patterns in the data revealed a different reality for most. Rather than encouraging engagement, such displays can even undermine the intervention’s purpose. When ED staff perceived these metrics as driven by managerial agendas, they felt their clinical autonomy was being compromised and that the complexities of frontline care were misunderstood. This perception can foster resentment and reduce engagement with the TC, which becomes associated with oversight and compliance rather than compassionate care (CMO15). Although run charts showing TC compliance and outcomes were not displayed in the ED, some staff expressed concern that such tools could be interpreted as tools for judgement rather than support, shifting the intervention’s focus away from reducing drug-related harm.

In contrast, narrative feedback—particularly when shared by outreach workers (OWs)—can powerfully reaffirm the value of the Trigger Checklist. When ED staff hear real-life case studies that illustrate how their actions have contributed to a person’s recovery journey, it reinforces the Trigger Checklist as a meaningful and effective tool. These stories evoke empathy, validate the clinician’s role in a broader continuum of care, and highlight the tangible impact of their efforts. This kind of feedback fosters a sense of purpose and connection, motivating staff to continue using the checklist not out of obligation, but from a genuine desire to support individuals at risk (CMO 16). Narrative feedback appeared to shift the perception of the checklist from a procedural task into a relational and meaningful intervention of value.

### Perception of less ED visits

When ED staff noticed that individuals with drug-related issues were attending less frequently, they attributed this change to the impact of the Trigger Checklist and associated outreach, reinforcing their belief in its value as a meaningful intervention rather than just a tick-box exercise (CMO 17). This shift in perception strengthens their motivation to use the checklist consistently, driven by a belief in its capacity to support recovery and reduce harm. It is worth noting that this was not driven by any formal statistics on attendance that ED staff had access to, but merely their own observations and anecdotal information. The initial CMO was revised to capture that the mechanism of reaffirming value to the person reflected the data better as compared to ED staff being hopeful that it would reduce the demand on the service.

## Discussion

The aim of this realist evaluation was to explore the reasoning behind emergency department (ED) clinicians’ responses to the Trigger Checklist, in order to understand why, how, and for whom the checklist could facilitate timely outreach for individuals at risk of drug-related harm attending a remote and rural ED. Analysis generated three programme theories: (1) confident decision-making to identify and refer; (2) trusting relationships between outreach workers (OWs) and ED staff; and (3) perceived value of the Trigger Checklist and its associated outreach. These theories were underpinned by the middle-range theory of relational coordination.

This study found that implementing the Trigger Checklist within a remote and rural ED can support the identification and assertive outreach of individuals most at risk of drug-related harm. However, its effectiveness is closely linked to the strength of the relationship between OWs and ED staff. We found that OWs are a critical component of the intervention pathway; in their absence, the utility of the Trigger Checklist in rural ED settings would likely be significantly reduced. In addition, relational coordination between the OWs and ED staff was foundational to the effective operationalising of the Trigger Checklist within the ED. For example, in time-pressured situations where there was uncertainty about whether completing the Trigger Checklist was appropriate, busy ED staff often chose not to due to the necessity to quickly attend to the next patient. However, when the OWs attended the ED to collect completed checklists, their presence acted as a visual prompt and created opportunities for case discussions with ED staff. OW shared their knowledge and experience of working with people in recovery, which informed the clinician’s decision as to the appropriateness of the referral.

The use of visual management tools [28] and human prompts [29] is well established in healthcare to support and direct decision-making. This study found that combining both the visual prompt with human interaction enhanced the likelihood of the ED clinician completing the Trigger Checklist. This is a critical finding, as it challenges assumptions around efficiency driven solutions, such as online referrals which would have undermined a key mechanism initiating Trigger Checklist completions. Specifically, removing the OWs from the process in favour of online submissions would likely compromise the intervention’s impact. Instead, the success of the checklist was contingent on strong interpersonal relationships, sustained through key aspects of communication; frequency, timeliness, accuracy, and problem-solving—core elements of relational coordination [26].

This study observed patterns of behaviour which identified that the Trigger Checklist was not a universal screening tool, like Alcohol Brief Interventions, but rather a tool to be used when relevant to the patient’s presentation. This aligns well with the principles of autonomous clinical decision-making and person-centred care. Evidence supports the assertion that checklists do aid, but do not replace clinical decision-making [30,31].

An initial programme theory proposed that including a statement reinforcing Caldicott Principle 7—emphasising the duty to share information when appropriate—might alleviate clinician anxiety around legal consequences. However, patterns in the data did not support this assumption. Instead, the key mechanism influencing use of the Trigger Checklist was clinicians’ underlying beliefs, particularly their fear of litigation and their confidence in being able to justify their decisions. This finding aligns with existing literature on defensive medicine, which shows that fear of legal repercussions often drives clinical behaviour more strongly than ethical guidance [32,33].

Data sharing and legal concerns continue to present significant barriers to connecting siloed services across health and social care [34–36]. Despite the existence of ethical frameworks such as the Caldicott Principles, clinicians often remain uncertain or anxious about the legal implications of sharing information, particularly in complex or high-risk cases. This persistent uncertainty underscores the need for more strategic and politically driven leadership to provide clear, consistent guidance and reassurance. Without such direction, frontline staff may default to risk-averse behaviours that hinder collaborative working and the integration of care. Addressing these barriers requires not only technical solutions but also cultural and systemic change—where legal literacy, interprofessional trust, and shared accountability are actively cultivated. Such an approach is consistent with relational coordination theory, which emphasises the role of shared goals, mutual respect, and effective communication in fostering confident and collaborative decision-making [26].

The findings from this study highlight the importance of trusting relationships between ED staff and OWs as a key mechanism enabling the effective use of the Trigger Checklist. When OWs possess high situational awareness, they can judge the right moment and approach for engaging ED staff, which is only possible when mutual respect and trust are present. This aligns with broader realist evaluation literature, which consistently identifies trust as a generative mechanism in inter-organisational and inter-professional collaborations. For example, Aunger et al [37] describe trust, confidence, and faith as foundational to knowledge sharing and joint decision-making across healthcare sectors. Similarly, realist evaluations of integrated care and first-contact physiotherapy services show that trust between staff from different departments or agencies facilitates role acceptance, adaptive behaviour, and improved service delivery. In this study, trust not only enabled knowledge exchange between OWs and ED staff but also created the conditions for collaborative discussion and action that ultimately benefited individuals at risk of drug-related harm. These findings suggest that trust-building should be an explicit focus in programme design and implementation, particularly where complexity and uncertainty are high where relational mechanisms are crucial for sustained engagement and impact.

This study found that ED staff were more inclined to complete the Trigger Checklist when they felt an intrinsic motivation to support individuals at risk of drug-related harm—particularly when they perceived the checklist as a meaningful tool that extended their impact beyond standard emergency care. Staff reported increased motivation when they observed reductions in repeat attendances and received narrative feedback from OWs about individuals who had been successfully supported. These findings suggest that intrinsic motivation—rooted in professional values, empathy, and a desire to make a difference—is a more powerful driver of engagement than extrinsic motivators such as performance metrics or data dashboards. In fact, few staff found data displays motivating; some even felt that an overemphasis on data diminished the perceived value of the intervention by shifting focus away from person-centred outcomes. This aligns with realist literature that emphasises the importance of aligning programme mechanisms with the internal reasoning and values of participants. For interventions like the Trigger Checklist to be sustained and effective, it is crucial to nurture intrinsic motivation through relational feedback, storytelling, and visible impact, rather than relying solely on quantitative indicators.

While this realist evaluation was conducted in a single remote and rural location, which may limit generalisability, the emphasis in realist methodology is on transferability. The findings may be applicable to similar contexts where comparable mechanisms and conditions exist. Realist theory development is inherently iterative, and further studies in diverse settings are needed to affirm, refine, or refute the programme theories developed here. In addition, the Trigger Checklist used to identify people at risk of drug harm was developed pragmatically through collaboration between Drug and Alcohol practitioners and individuals with lived experience. Formal evaluation of the checklist’s sensitivity and specificity was beyond the scope of this work. Although all referrals were reviewed by a Drug and Alcohol Practitioner prior to outreach and very few were subsequently deemed inappropriate, future research should formally examine the performance characteristics of the checklist and explore how different referral thresholds may influence identification and engagement.

A further limitation of this study is that the final programme theories were not reviewed by the Project Advisory Group or study participants due to time constraints and service pressures. However, they were iteratively refined using data from those delivering and experiencing the intervention throughout the project. Informal feedback following dissemination of the findings to practitioners and individuals supporting Drug and Alcohol services indicated that the findings resonated with their experiences. Nevertheless, further formal stakeholder input would strengthen confidence in the proposed context–mechanism–outcome explanations and should be incorporated in future studies.

## Conclusion

This study provides theory-informed insights into implementing a pathway that connects individuals at risk of drug-related harm in EDs with recovery services. By identifying key context–mechanism–outcome patterns, it deepens understanding of how relational and contextual dynamics shape frontline implementation. Relational coordination emerged as a critical aspect to bridge disconnected services and enable timely referrals. These findings confirm and extend existing theory on inter-service collaboration and can inform future adaptations and scaling strategies that remain responsive to local conditions and staff reasoning.

## Supporting information

S1 File Initial CMOs to final CMOs. (DOCX)

S2 File Topic guide for stakeholders interview. (DOCX)

S1 Fig Conceptual framework. (TIF)

S1 Table CMO configurations and exemplar quotations. (DOCX)
