# Supplementary material for: Implementation of the Trigger Checklist and assertive outreach to reduce drug-related harm in the Emergency Department: A realist evaluation
Source: PLoS One. 2026 Oct 5;21(10):e0358880. doi: 10.1371/journal.pone.0358880 (PMC13637928; doi:10.1371/journal.pone.0358880)
Supplement: Supplementary file 1 [file pone.0358880.s001.docx]

S1 Appendix: Initial CMOs to Final CMOs

| **INITIAL CMOs** | **ALETRATION** | **NEW CMOs** |
| --- | --- | --- |
| **Programme theory 1: Confident decision-making** | | |
| **Certainty (CMO 1,2 & 3)** | | |
| If the person who is using drugs is seriously ill in the ED (classed as major or in resuscitation area) then clinical staff (nurses and doctors) will be less likely to complete the Trigger Checklist as the seriousness of the patient’s physical condition prioritises urgent action and staff forget to complete it | Retain | 1. If the person who is using drugs is seriously ill in the ED (classed as major or in resuscitation area) then clinical staff (nurses and doctors) will be less likely to complete the Trigger Checklist as the seriousness of the patient’s physical condition prioritises urgent action and staff forget to complete it. |
| Rival  If the person who is using drugs is seriously ill related to their drug use in the ED (classed as major or in resuscitation area) then ED staff will be more likely to complete the Trigger Checklist as the seriousness of the patient’s condition creates a call for action and their altruistic desire to help the person triggering ED staff to prioritise completion of the Trigger Checklist | Remove |  |
| If ED staff perceive that the patient is seriously injured and presentation is assault (which could be drug-related- then clinical staff (nurses and doctors) will not complete the Trigger Checklist as they are unsure if appropriate and presume that if they are still using drugs then the recovery team will already know about them. | Revise | 2. If the person presents with a high suspicion of drug-related illness or injury or the person is known to use drugs (either from frequent ED attendances or known in the rural community as someone who takes drugs) then ED staff are more likely to complete the Trigger Checklist as they are confident as to the appropriateness of the referral and believe the support from outreach will be of value. |
|  | New | 3. If the person who uses drugs is known by the ED staff and has an increase in frequency in ED attendance, presents in a more unwell state (physical or mental) than their usual presentation, or they appear vulnerable, then ED staff are more likely to complete the Trigger Checklist, as ED staff are concerned for the individual and perceive the presentation as an escalation in the persons drug use and risk of harm. |
| **Aid to conversation (CMO 4)** | | |
|  | New | 4. When ED staff are familiar with the Trigger Checklist and have built a rapport with the person using drugs over time due to frequent attendances they use the 10 additional questions on the Trigger Checklist questions to focus their interaction person around the drug-related harm and the person using drugs often provides the answers without the questions being asked directly as ED staff feel this approach will not damage the therapeutic relationship and is a more natural patient interaction |
| **Confidentiality breach versus harm (CMOs 5&6)** | | |
| If the checklist details on the form that there is no need for patient consent to complete the Trigger Checklist, then they will complete it as this allays their fears and anxieties around breaching patient confidentiality and getting into trouble legally. | Revised | 5. If ED staff understand and believe that Caldicott Guardianship Principle 7 (the duty to share information for individual care is as important as the duty to protect confidentiality) then they use confident clinical decision-making to complete the TC  as they can justify disclosure when the level of patient vulnerability and risk of harm outweighs any concern of reprisal or fear of litigation for sharing patient confidential information with the recovery service. |
| If ED staff feel that they should not share the responses from the TC with the DARS team without the patients' consent they will not complete the Trigger Checklist for fear of damaging the therapeutic relationship with the patient and fear of litigation for sharing personal information. | Revised | 6. If ED staff believe that patient confidentiality it sacrosanct and they are not aware of the updates Caldicott Guardianship Principles then they will not complete the Trigger Checklist, without the patents consent, even if there is a statement on the TC that specifies no need for consent, for fear of breaching patient confidentiality with resultant damage to the therapeutic relationship and possible litigation. |
| **Regular human prompt (CMO 7)** | | |
|  | New | 7. When the outreach workers visit twice daily to collect any referrals this initiates ED staff to complete a Trigger Checklist or, at least discuss a potential referral, as the visual presence of the outreach worker triggers a reminder for busy ED staff and the frequency of visits enables ED staff to have a timely discussion in cases where they are uncertain about the appropriateness of the referral.   The outreach worker shares their knowledge of drug recovery and people known to their service which shifts their uncertainty to confident decision making. |
| **Programme theory 2: Trusting relationship** | | |
| **Approachable recovery outreach workers (CMO 8)** | | |
|  | New | 8. When the outreach workers interact in a caring, non-judgemental way with both the people who use drugs and the ED staff this encourages the ED staff to approach the outreach workers with any questions as they trust the outreach workers to advise them and their conversation with the outreach worker triggers compassion for the patient  which encourages a non-judgemental approach and initiates a desire to help people who present with problems associated with drug use. |
| **Situational awareness of recovery outreach workers (CMO 9)** | | |
|  | New | 9. When the recovery outreach workers interact in a caring, non-judgemental way with both the people who use drugs and the ED staff this encourages the ED staff to approach the outreach workers with any questions as they trust the Outreach Workers to advise them and their conversation with the Outreach Worker triggers compassion for the patient which encourages a non-judgemental approach (M) which initiates a desire to help people who present with problems associated with drug use. |
| **Trust (CMO 10 & 11)** | | |
| If ED staff feel that asking patients whether they use drugs is offensive they will not complete the Trigger Checklist for fear of patients' distrust leading to them not accessing care in the ED in the future | Part combined with New | 10. If the person presenting with a drug problem is fearful of the potential outcomes of answers they give to the TC then they will not give an honest response for either shame and fear of being judged for their drug taking behaviour, fear of ED staff telling their family or others known to them or for reporting them to police or social services because they mostly distrust public/government funded services.  However, this fear can be mitigated when the OW are present in the ED, or over time as the person using drugs builds a relationship with the ED staff which gives the person the confidence and trust to disclose honestly. |
|  | New | 11. In a close-knit rural community, a professional relationship between the OW and ED staff takes time to develop and legitimises them both to disclose sensitive patient information for the benefit of the person at risk of drug-related harm as both feel confident to share information and trust each other that the information will not be shared out with the ED or recovery service. |
| **Mutual respect (CMO 12)** | | |
|  | New | 12. When  ED  staff and outreach worker mutually respect each other’s roles  it enables them to have two-way knowledge exchange and reaffirms each other’s value which enables the best outcome for the patient (physical care whilst in the ED and follow up-outreach). |
| **Programme theory 3: Perceived value** | | |
| **Altruism (CMO 13 & 14)** | | |
| If staff in the rural ED have witnessed the serious outcome of individuals who use drugs i.e. death or non-fatal overdose, then they will complete the Trigger Checklist for others who use drugs and attend the ED because the emotional impact of the previous event on themselves and the wider community motivates them to do whatever they can to prevent something similar reoccurring. | Revised | 13. If staff in the rural ED have experienced the serious outcome of individuals who use drugs i.e. death or non-fatal overdose, then they will complete the Trigger Checklist for others who use drugs and attend the ED because the emotional impact and associated empathy of the previous event on themselves and the wider community motivates them to do whatever they can to prevent something similar reoccurring and remind them of the value of completing the checklist. |
|  | New | 14. If staff in the rural ED know and have access to a process which enables then to refer  individuals who use drugs  that are at risk of harm, then they will complete the Trigger Checklist for others who use drugs and attend the ED because they feel empathy and a desire to help the person and acknowledge that the need goes beyond what is available within an ED. |
| **Value of feedback (CMO 15 & 16)** | | |
| If data on the Trigger Checklist use and outcomes is displayed visually in the ED where metrics are used for improvement, then staff will complete it because it is a visual reminder for busy ED staff and it motivates them to perform well due to competitiveness with other clinical areas or for fear of punitive repercussions. | Revised | 15. If measurement/stats of the Trigger Checklist are perceived as being displayed in the ED because of a  ‘push by management’ then ED staff will be less likely to use it, because they feel resentful that their situation in the ED is not being understood, that they do not have the freedom to use their own clinical decision-making  and, that it is another tool for judgement or management performance as opposed to the potential value of improving the lives of those most at risk of drug related harm. |
| If the outreach workers feedback positive  Outcomes via storytelling or cases they have successfully outreached to the ED staff, then they will be motivated to prioritise and complete the Trigger Checklist because the stories incentivise them. | New | 16. If the outreach workers feedback positive narrative case studies of outreaching those at risk of harm to ED staff, then the value of the Trigger Checklist is reaffirmed, and ED staff feel that they are making a difference, it is beneficial and worthy of their time to complete it  as patients are followed up and potentially supported to recover and ED staff are motivated to complete the Trigger Checklist in the future. |
| **Perception of less ED visits (CMO 17)** | | |
| If ED staff think the trigger Checklist and associated outreach will reduce attendance of those with drug related harm, they will be more likely to use it as they are hopeful the person will get the help they need and believe it to be worthwhile and potentially reduce some of the pressure from the high demand on their service as people who use drugs are often frequent attenders. | Revised | 17. If ED staff perceive that previous frequent attenders with drug-related issues now visit the ED less frequently then, they are more likely to use the Trigger Checklist as the reduced attendances validates and reaffirms the value in the tool and associated outreach. |
| **CMOs Deleted** |  |  |
| If ED staff who would be completing the Triger Checklist are engaged in shaping the ED Trigger Checklist pathway, then they are more likely to complete the Trigger Checklist as they feel a sense of influence and ownership for the change to their practice. | Deleted |  |
| If the Trigger Checklist has a limited amount of easy to answer questions and an open-ended text box, then ED staff will be more likely to complete it and it is of use to the outreach workers due to the limited cognitive load, ease of the administrative burden and freedom to share other relevant information which is not in response to the set questions. | Deleted |  |
| If the checklist details on the form that there is no need for patient consent to complete the Trigger Checklist, then they will complete it as this allays their fears and anxieties around breaching patient confidentiality and getting into trouble legally. | Deleted |  |
| If ED staff receive training which includes understanding that completing the Trigger Checklist will initiate assertive outreach, which can save a person’s life, then they will complete it because ED staff prioritise emergency life saving care. | Deleted |  |
| If ED staff believe that people who use drugs cannot recover, then they will not complete the Trigger Checklist as their fatalistic belief suggests outreach will not help and influences their behaviour of non-completion. | Deleted |  |
| If ED staff are unsure where their responsibilities lie in relation to responses given from the TC then they will not ask the questions as doing so could include social work and or child welfare concerns which creates fear, uncertainty and worry for ED staff around associated ethical dilemmas and litigation concerns. | Deleted |  |
| If ED staff feel unskilled to ask patients whether they use drugs they will not complete the Trigger Checklist for both fear of offending the patient and damaging the therapeutic relationship and feeling apprehensive/anxious about not responding appropriately to what the patient shares. | Deleted |  |
| If ED staff are trained to ask and respond to difficult questions around drug use, then they will complete the Trigger Checklist as they will feel confident and not fear causing offence and/or upset to the person who uses drugs. | Deleted |  |
| If ED staff are from other clinical areas or on the bank, they will not complete the Trigger Checklist as they will not have knowledge that completing the TC is required. | Deleted |  |
| If ED staff (doctors, nurses and HCAs) think it is not their role to identify those most at risk of drug related harm, then they will not complete the Trigger Checklist as they feel overwhelmed and resentful that this task is being added to their already demanding workload. | Deleted |  |
| If the Trigger Checklist is supported by senior clinicians, then ED staff are more likely to complete it as leadership endorsement both legitimises the checklist completion in the hierarchical culture of healthcare and appeases those in senior positions. | Deleted |  |
| If the Project Lead is influential and respected, then ED staff are more likely to engage with the project as staff will view the idea as credible. | Deleted |  |
