# Supplementary material for: Implementation of the Trigger Checklist and assertive outreach to reduce drug-related harm in the Emergency Department: A realist evaluation
Source: PLoS One. 2026 Oct 5;21(10):e0358880. doi: 10.1371/journal.pone.0358880 (PMC13637928; doi:10.1371/journal.pone.0358880)
Supplement: Supplementary file 2 [file pone.0358880.s002.docx]

**Reducing Drug-Related Harm to Outreach Proactively from**

**Emergency Care (DR_HOPE Project)**

**Topic Guide for Stakeholders Interview**

| Introduction | - Principle investigator - UHI Researcher - Confirm read PIS, happy to proceed - Any questions before we start |
| --- | --- |
| Consent | - Start recording and reconfirm consent |
| Ice breaker | - Tell me about your experience in the ED/Outreach team of working with people who use drugs |
| Introduce IRPTS | - Explain how teacher/learner cycle works - Emphasise we are testing theories – agree, disagree, change them - No right or wrong answers - 8-10 theories to test |
| Probe for mechanisms | - “what is about about using the Trigger Checklist that worries you…… - “Do you think X changed how you felt… - “Do you think you would have reacted differently if...” - “What worries you about using the Trigger Checklist?” |
| Context | - What is the perception of people who use drugs by ED staff generally? - Does the seriousness/reasons of the presentation influence whether you would complete the Trigger Checklist or not? - Is there anything about living in remote/rural location that impact on how people using drugs are identified and outreached in the ED? - Leadership – what’s it like here? - Experience of those who use drugs – influence perceptions? Action to trigger and refer? |
| What works, for whom | - Who do you think would get the most benefit from being referred (all patients, those known to use drugs, those who want help now? - Who would be best to complete the Trigger Checklist? - What would make it easy to complete? |
| Disconfirming cases/rivalry | - Unintended consequences… “could this backfire?” - Present alternate theories/contradictory theories – “For nurses X but for doctors Y” - “Other stakeholders have suggested….” - “The theory behind the policy suggested X but….” |
