# Supplementary material for: Implementation of the Trigger Checklist and assertive outreach to reduce drug-related harm in the Emergency Department: A realist evaluation
Source: PLoS One. 2026 Oct 5;21(10):e0358880. doi: 10.1371/journal.pone.0358880 (PMC13637928; doi:10.1371/journal.pone.0358880)
Supplement: Supplementary file 3 [file pone.0358880.s004.docx]

Table 1: CMO & Exemplar Quotations

| **CMO** | **Participant** | **Exemplar quotation** |
| --- | --- | --- |
| **Programme theory 1: Confident decision-making** | | |
| 1. If the person who is using drugs is seriously ill in the ED **(C)** then clinical staff will be less likely to complete the Trigger Checklist **(O)** as the seriousness of the patient’s physical condition prioritises urgent action and staff forget to complete it **(M).** | P1, P2, P6, P10 | *“Maybe they even need intubation because their airway is compromised. Obviously, I’m not going to prioritise the checklist - I have to be honest.”* P6 |
| 2. If the person presents with a high suspicion of drug-related illness or injury or the person is known to use drugs **(C)** then ED staff are more likely to complete the Trigger Checklist **(O)** as they are confident as to the appropriateness of the referral and believe the support from outreach will be of value **(M)**. | P4, P7, P13 | *“But unless there was a strong thought there had been drugs taken by the person, by their presentation, we wouldn’t Trigger it just on an assault unless we knew their history and they’d benefit.”* P4 |
| 3. If the person who uses drugs is known by the ED staff and has an increase in frequency in ED attendance, presents in a more unwell state (physical or mental) than their usual presentation, or they appear vulnerable **(C**), then ED staff are more likely to complete the Trigger Checklist **(O),** as ED staff are concerned for the individual and perceive the presentation as an escalation in the persons drug use and risk of harm **(M)**. | P3, P8, P9, P12 | *“I think if a young person comes in with anything like that (drug use), it does give you more of a red alert because you feel that that young person is quite vulnerable, especially if they don’t have any sort of help with their parents and things like that, so you do feel like there’s a little bit more urgency.” P3* |
| 4. When ED staff are familiar with the Trigger Checklist and have built a rapport with the person using drugs over time due to frequent attendances **(C)** they use the 10 additional questions on the Trigger Checklist to have a drug-related harm conversation and the person using drugs often provides the answers without the questions being asked directly **(O)** as ED staff feel this approach will not damage the therapeutic relationship and is a more natural patient interaction **(M)**. | P3, P5, P7, P12, P13 | *“I’ll judge the situation and try and build a rapport with the person, make them see that we’re here to help, that they can trust us, so that it’s not like –‘well you need to tell us, this, this and this’, sort of thing.” P7* |
| 5. If ED staff understand the duty to share information for individual care is as important as the duty to protect confidentiality) **(C)** then they complete the TC without the patient’s consent **(O)** as their concern for the patient overrides any concern of reprisal for sharing patient confidential information with the recovery service **(M)**. | P4, P6, P11 | *“I think if it was a young, vulnerable lassie, I’d be inclined to – if she’d come in a couple of times, maybe I would just feel, for her benefit, I would do it, yeah, without asking. If she was a lassie that was easily influenced and maybe getting taken advantage of by people.”* P11 |
| 6. If ED staff believe that patient confidentiality is sacrosanct and they are not aware of the Caldicott Guardianship Principles **(C)** then they will not complete the Trigger Checklist without the patents consent **(O)** for fear of possible litigation for breach of patient confidentiality or damage to the therapeutic relationship **(M)**. | P2, P11, P12 | *“Where they’ve said ‘no’ but clinical observation says something else, and I’ve asked the question and they’ve gone, ‘No, I don’t want that,’ I feel like that would damage the trust of the patient if I then went ahead and did it anyway, so if they then, later on, got a follow-up from the service, next time they might not come to ED.” P12* |
| 7. When the recovery outreach workers visit twice daily to collect any referrals **(C)** this initiates ED staff to complete a Trigger Checklist or, at least discuss a potential referral **(O)**, as the visual presence of the outreach worker triggers a reminder for busy ED staff **(M)** and the frequency of visits enables ED staff to have a timely discussion in cases where they are uncertain about the appropriateness of the referral **(M)**.   The outreach worker shares their knowledge of drug recovery and people known to their service which shifts their uncertainty to confident decision making **(O)**. | P2, P4, P5, P8, P10, P11, P12 | *“They’re a human prompt rather than a visual prompt, it’s an inter-personal thing, having a visual prompt can get ignored as well.”* P10  *“They’ll come in and they’ll pick the forms up, if there’s forms there, then they’ll say, ‘did anyone come in who you weren’t sure?”* 5 |
| **Programme theory 2: Trusting relationships** | | |
| 8. When the recovery outreach workers interact in a caring, non-judgemental way with both the people who use drugs and the ED staff **(C)** this encourages the ED staff to approach the outreach workers with any questions **(O)** as they trust the Outreach Workers to advise them **(M)** and their conversation with the Outreach Worker triggers compassion for the patient  which encourages a non-judgemental approach **(M)** which initiates a desire to help people who present with problems associated with drug use **(O)**. | P3, P4, P5, P6, P8, P11 | *“I think it does make a difference (a good relationship with the Outreach Worker), yeah. Yeah. No, it definitely does make a difference because we have watched them and it’s that ripple effect, it gives that – a good rapport, if they are comfortable coming into our Department, we are comfortable with them being there. And obviously then that ripples onto the patient.” P3* |
| 9. If the ED is very busy and names on the whiteboard or database are known to the recovery service **(C)** then the ROW use situational awareness **(M)** to judge when to have a conversation with the ED staff regarding whether the Trigger Checklist is needed **(O)** and/or they accept a partially completed Trigger Checklist **(O)**. | P1, P2, P13 | “*Well, we understand, they are quite often rushed for time, and we’d rather get the form with no additional information than not get it at all.” P2* |
| 10. If the person presenting with a drug problem is fearful of the potential outcomes of answers they give to the TC **(C)** then they will not give an honest response **(O)** for either shame and fear of being judged for their drug taking behaviour **(M)**, fear of ED staff telling their family or others known to them **(M)** or for reporting them to police or social services because they mostly distrust public/government funded services **(M).**  However, this fear can be mitigated when the OSW is present in the ED, or over time as the person using drugs builds a relationship with the ED staff **(C)** which gives the person the confidence and trust (M) to disclose honestly **(O)**. | P3, P7, P8, P11 | *“I find a lot of the time though; they don’t want to engage because they are ashamed. They are, they are ashamed. And I’m mainly meaning, not only with drugs but with alcohol and they hide it and say they take less units, they take less drugs. And it’s because they are really ashamed. And then you get X (Outreach Worker) coming in and they are so friendly and non-judgemental, that really helps them open up.”* P11 |
| 11. In a close-knit rural community, a professional relationship between the Outreach Worker and ED staff takes time to develop **(C)** and legitimises them both to disclose sensitive patient information **(M)** for the benefit of the person at risk of drug-related harm **(O)** as they develop confidence to share information and trust each other that the information will not be shared out with the ED or recovery service **(M).** | P4, P8, P12 | “*So, you are sharing information between yous, between the different services. But I don’t think…obviously there’s trust between, we are giving them information and they are giving us information. But we’re all covered by the same confidentiality, so it’s not like they are going to go and spread the news and we’re not going to spread the news that such and such is taking drugs.”* P4 |
| 12. When ED staff and outreach workers mutually respect each other’s roles **(C)** it enables them to have two-way knowledge exchange and reaffirms each other’s value **(M)** which enables the best outcome for the patient - physical care whilst in the ED and follow up-outreach by DARS **(O)**. | P1, P4, P5, P7 | “And we are asked things most weeks like. And doctors, I’ve had doctors come up to me. ‘X , we've held back because we wanted to ask you something.’ And that's fine. Good God, a band 3 and that's by a doctor.” P1 |
| **Programme theory 3: Perceived value** | | |
| 13. If staff in the rural ED have experienced serious outcomes among individuals who use drugs, such as a death or non-fatal overdose **(C)**, then they will complete the Trigger Checklist for others presenting with drug-related issues **(O)** because of the emotional impact of the event and a deepened sense of empathy for both the individual and wider community, motivates staff to take steps to reduce something similar reoccurring and remind them of the value of completing the checklist **(M)**. | P7, P8, P9, P12, P13 | *“I remember … sitting on the pavement outside the hospital with a young lad and I said to him, ‘why do you do this? Why do you keep doing it?’ And he said to me, ‘because nobody gives a F about me, and I don’t care if I live or die.’ And do you know, it was heart-breaking, it took me everything not to sit and cry. And we sat and we laughed, and we joked and a week later … ‘they’ve just found him dead.’ He was seventeen”*  *“I think because, again with it being a small place, if you are on shift when something like that happens, it’s in the back of your mind and you would think if they’d been in previously, if they’d been in a couple of months before that, if a Trigger Checklist had been done, would it have happened? …  You just don’t want to miss anyone.”  P13* |
| 14. If staff in the rural ED are aware of and have access to a clear process (completing the Trigger Checklist) that enables them to refer individuals at risk of drug-related harm **(C)**,  then they will complete the Trigger Checklist **(O)** because they feel empathy and a desire to support the person and provides them with a meaningful intervention **(M)** which is usually limited in an ED. | P5, P6, P8, P12 | *“You don’t want to just brush them under the carpet, that they’ve been in, we’ve dealt with them because really you know that’s not going to actually help them in the long run, they need a bit more support in the community.”* P8 |
| 15. If measurement/stats of the Trigger Checklist are perceived as being displayed in the ED because of a  ‘push by management’ **(C)** then ED staff will be less likely to use it **(O)**, because they feel resentful that the realities of working in ED are not understood, undermines clinical autonomy, and positions the Checklist as a tool for judgement or management performance rather than as a meaningful intervention to support individuals at risk of drug-related harm **(M)**. | P1, P4, P5, P11 | “*Yes, I think, going back to our waiting room, I remember going in it and it was all laminated sheets with all these different run charts but X used to do it all for us, and I was thinking it’s all about charts now,  it does tend to take over rather than the importance of care and what the outcome is.”* P11 |
| 16. When Outreach Support Workers share positive narrative case studies with ED staff **(C)** then the value of the Trigger Checklist is reaffirmed, and ED staff feel that their actions are making a tangible difference, that completing the checklist is worthwhile, and that patients are being followed up and supported in their recovery which  motivates staff **(M)** to complete the Trigger Checklist in the future **(O)**. | P2, P3, P4, P7, P8, P9, P10. | *“Yeah, or the outreach worker saying, as I say, ‘Oh I’ve been trying to get this person to engage for a long time and we’ve managed to get them to engage’, but just because you’ve done this trigger and this further attempt has made a difference, when you are trying to get this person for a long time, to engage. So, a positive story like that can push its use.”* P4 |
| 17. If ED staff perceive that previous frequent attenders with drug-related issues now visit the ED less frequently **(C)** then, they are more likely to use the Trigger Checklist **(O)** as the reduced attendances proves and reaffirms the value in the tool and associated outreach **(M)**. | P1, P4 | *“So, you notice that there’s good things coming from it because they are interacting with the team and they are keeping them on an even keel. Like people we’ve had in for years, some of them, I haven’t seen for…since the trigger checklist came into the department, they haven’t been attending. Whereas before, they were in once a week, at least, if not more. So, something has gone right in the drugs and alcohol team, something is going right.”* P4 |
