# Supplementary material for: Implementation of the Trigger Checklist and assertive outreach to reduce drug-related harm in the Emergency Department: A realist evaluation
Source: PLoS One. 2026 Oct 5;21(10):e0358880. doi: 10.1371/journal.pone.0358880 (PMC13637928; doi:10.1371/journal.pone.0358880)
Supplement: Supplementary file 4 [file pone.0358880.s006.doc]

Ass Prof Michelle Beattie

Centre for Rural Health Sciences

University of Highlands and Islands

UHI House

Old Perth Road

Inverness

IV2 3JH

7^th^ August 2026

Dear Professor Berghs

Manuscript ID PONE-D-25-67347 Implementation of the Trigger Checklist and Assertive Outreach to Reduce Drug-Related Harm in the Emergency Department: A Realist Evaluation

Thank you very much for coordinating the further revision of our manuscript, which we hope will be considered for publication in PLOS ONE.

Please find details of responses to reviewer’s and editorial comments below.

| Editorial Comments | Response |
| --- | --- |
| 1.Please ensure that your manuscript meets PLOS ONE's style requirements, including those for file naming. The PLOS ONE style templates can be found at  https://journals.plos.org/plosone/s/file?id=wjVg/PLOSOne_formatting_sample_main_body.pdf and  https://journals.plos.org/plosone/s/file?id=ba62/PLOSOne_formatting_sample_title_authors_affiliations.pdf | All formatting updated in accordance with the Manuscript Body Formatting Guidelines and Title, Author, Affiliations Formatting Guidelines. |
| 2. Please note that funding information should not appear in any section or other areas of your manuscript. We will only publish funding information present in the Funding Statement section of the online submission form. Please remove any funding-related text from the manuscript. | The Funding Statement has been removed from the manuscript. |
| 3. Thank you for stating the following financial disclosure:  “This study was funded through Q Exchange by the Health Foundation and NHS England”  Please state what role the funders took in the study. If the funders had no role, please state: "The funders had no role in study design, data collection and analysis, decision to publish, or preparation of the manuscript."  If this statement is not correct you must amend it as needed.  Please include this amended Role of Funder statement in your cover letter; we will change the online submission form on your behalf. | This study was funded through Q Exchange by the Health Foundation and NHS England (statement removed from main manuscript).  Role of Funder for online submission “The funders had no role in study design, data collection and analysis, decision to publish, or preparation of the manuscript.” |
| 4. We note that you have indicated that there are restrictions to data sharing for this study. PLOS only allows data to be available upon request if there are legal or ethical restrictions on sharing data publicly. For more information on unacceptable data access restrictions, please see http://journals.plos.org/plosone/s/data-availability#loc-unacceptable-data-access-restrictions.  Before we proceed with your manuscript, please address the following prompts:  a) If there are ethical or legal restrictions on sharing a de-identified data set, please explain them in detail (e.g., data contain potentially identifying or sensitive patient information, data are owned by a third-party organization, etc.) and who has imposed them (e.g., a Research Ethics Committee or Institutional Review Board, etc.). Please also provide contact information for a data access committee, ethics committee, or other institutional body to which data requests may be sent.  b) If there are no restrictions, please upload the minimal anonymized data set necessary to replicate your study findings to a stable, public repository and provide us with the relevant URLs, DOIs, or accession numbers. For a list of recommended repositories, please see  https://journals.plos.org/plosone/s/recommended-repositories. You also have the option of uploading the data as Supporting Information files, but we would recommend depositing data directly to a data repository if possible.  We will update your Data Availability statement on your behalf to reflect the information you provide. | Although the dataset was anonymised prior to analysis, there remains a potential risk of participant re-identification due to the small size of the rural communities included in the study. Several participants occupy specific professional or community roles that are held by one or a small number of individuals within these communities. As a result, the sharing of even de-identified qualitative data could inadvertently enable identification of participants when combined with contextual information.  The study was conducted under ethical approval granted by the University of the Highlands and Islands Ethics Committee (ID: ETH2425-0561), and participants provided informed consent on the understanding that their information would remain confidential. The approved participant information and consent form stated:  *“I understand that any information recorded in the interview will remain confidential and no information that identifies me as an individual will be made publicly available.”*  Making the dataset publicly available would therefore be inconsistent with the assurances of confidentiality provided to participants and approved by the Ethics Committee. For these ethical reasons, we do not consider public sharing of the dataset to be appropriate.  If you have any further questions or wish to contact an independent person to whom any questions may be directed, please contact:  UHI Research Ethics Officer  University of the Highlands and Islands Research Ethics Committee (REC)  University of the Highlands and Islands, 12b Ness Walk, Inverness, IV3 5SQ.  Telephone: 00 441463-279349  Email: research.ethics@uhi.ac.uk |
| 5. If the reviewer comments include a recommendation to cite specific previously published works, please review and evaluate these publications to determine whether they are relevant and should be cited. There is no requirement to cite these works unless the editor has indicated otherwise. | No recommendation to cite specific work in reviewers’ feedback. |
| 6. Please review your reference list to ensure that it is complete and correct. If you have cited papers that have been retracted, please include the rationale for doing so in the manuscript text, or remove these references and replace them with relevant current references. Any changes to the reference list should be mentioned in the rebuttal letter that accompanies your revised manuscript. If you need to cite a retracted article, indicate the article’s retracted status in the References list and also include a citation and full reference for the retraction notice. | References are complete and correct. The figure has been reformatted to meet the journal requirements using NASS. |
| To ensure your figures meet our technical requirements, please review our figure guidelines: https://journals.plos.org/plosone/s/figures  You may also use PLOS’s free figure tool, NAAS, to help you prepare publication quality figures: https://journals.plos.org/plosone/s/figures#loc-tools-for-figure-preparation.  NAAS will assess whether your figures meet our technical requirements by comparing each figure against our figure specifications. |  |
| Reviewer 1 Comments | Reviewer 1 Responses |
| The checklists relative simplicity and low threshold (or high sensitivity) are to be commended, though it would be useful to know if the investigators attempted any form of sensitivity analysis (even in the abstract where the likely impact of increasing and deceasing sensitivity might be considered). The extrapolation that 3/4s of those using drugs in the area were not known engaged with services served to further highlight the importance of the project. | We thank the reviewer for this observation. The Trigger Checklist was developed pragmatically through collaboration between experienced Drug and Alcohol practitioners and individuals with lived experience of recovery, with the primary aim of maximising identification of potentially vulnerable individuals who may benefit from outreach. A formal sensitivity analysis was not undertaken as part of this service evaluation/project, and we acknowledge this as a limitation. However, all completed Trigger Checklists were reviewed by a qualified Drug and Alcohol Practitioner prior to outreach activity to assess appropriateness and risk. Practitioner feedback indicated that only a very small number of referrals (fewer than five cases during the project) were subsequently considered unsuitable for outreach. While these observations do not constitute a formal assessment of sensitivity or specificity, they suggest that the checklist functioned as intended in identifying individuals potentially in need of engagement. We have added text to the manuscript acknowledging the absence of formal sensitivity analysis and recommending this as an important area for future evaluation of the checklist (see lines 657-665). |
| Having refined their methods in the course of looking at this setting therefore the authors are well placed to look at generalisability to other settings and other regions (e.g. the larger central belt populations), where the stigma of limited anonymity is less prevalent, but would the relative patient numbers result in a prohibitive work-load in the short term? | We thank the reviewer for this observation. From a realist perspective, we would not necessarily expect the same context–mechanism–outcome configurations to operate in different settings. Rather, future work would test whether mechanisms identified in this study, such as the value of relational outreach, trust-building, and concerns regarding information sharing, remain important under different contextual conditions. While issues relating to anonymity and stigma may be less pronounced in larger urban populations, alternative contextual influences may become more important.  We also agree that feasibility warrants exploration. The continuation of the service in this area was facilitated through organisational reconfiguration and the subsequent creation of two outreach worker posts. Urban areas may face different workforce and capacity challenges due to higher referral volumes, making evaluation of scalability and resource requirements an important next phase of testing and refinement. A quality improvement project is currently underway to test implementation of the Trigger Checklist within an urban Emergency Department setting, which may provide valuable insights into capacity requirements and inform our next realist study. |
| Reviewer 2 Comments | Reviewer 2 Responses |
| 3. Have the authors made all data underlying the findings in their manuscript fully available?  The PLOS Data policy requires authors to make all data underlying the findings described in their manuscript fully available without restriction, with rare exception (please refer to the Data Availability Statement in the manuscript PDF file). The data should be provided as part of the manuscript or its supporting information, or deposited to a public repository. For example, in addition to summary statistics, the data points behind means, medians and variance measures should be available. If there are restrictions on publicly sharing data—e.g. participant privacy or use of data from a third party—those must be specified. | See response to point 4 of the editorial comments. |
| 1. The use of a Project Advisory Group to develop initial programme theories was a strength – was this group consulted to comment on refined/final theories? (Or perhaps the study participants?) If so, please could this be made clear in the paper, and if not, I suggest you discuss this as a limitation. Confirmation from those who understand the lived experience of the intervention is important in realist work and would strengthen the credibility of conclusions. | We thank the reviewer for this important observation. The Project Advisory Group was involved in the development and refinement of the initial programme theories; however, the refined and final programme theories were not subsequently presented to the group. This was primarily due to time constraints within the project and challenges securing participation from clinical staff given competing service demands. We agree that stakeholder validation of refined programme theories can enhance the credibility of realist findings and provide an opportunity to assess the resonance of interpretations with lived and professional experience. We have therefore acknowledged the absence of final theory refinement with the Project Advisory Group as a limitation and recommend this as an important component of future realist evaluations. See lines 666-674 in the discussion section. |
| 2. I note the team is (appropriately) a mix of clinical and academic staff. Given the number of subjective decisions necessary within such a review (especially during testing the CMOs), you might consider a short section on team reflexivity to help the reader situate the analysis and aid transparency about how the researchers reduced bias and used reflexive practice in the analytical process. | We thank the reviewer for this valuable suggestion. Reflexive discussions were embedded throughout the analysis, although this was not sufficiently described in the manuscript. The multidisciplinary team, comprising academic and clinical members, met weekly during analysis to discuss emerging findings, challenge interpretations, and refine programme theories and CMO configurations. Particular emphasis was placed on retroductive reasoning, with team members debating plausible underlying mechanisms and alternative explanations for observed outcomes. These discussions supported reflexivity, consensus-building, and consideration of different professional perspectives. We have now added a short reflexivity section to the manuscript (see line 276-283 in methods). |

We are grateful for the feedback and believe it has improved our paper.

We look forward to hearing from you.

Sincerely,

Dr Michelle Beattie

On behalf of DR HOPE Team
