# Supplementary material for: Implementation of the Trigger Checklist and assertive outreach to reduce drug-related harm in the Emergency Department: A realist evaluation
Source: PLoS One. 2026 Oct 5;21(10):e0358880. doi: 10.1371/journal.pone.0358880 (PMC13637928; doi:10.1371/journal.pone.0358880)
Supplement: Peer review history

# Peer review: Decision Letter 0

**DOI:** 10.1371/journal.pone.0358880.r001

**Authors:** Maria Berghs

7 Jul 2026

Implementation of the Trigger Checklist and Assertive Outreach to Reduce Drug-Related Harm in the Emergency Department: A Realist Evaluation

PLOS One

Dear Dr. Beattie,

Thank you for submitting your manuscript to PLOS ONE. After careful consideration, we feel that it has merit but does not fully meet PLOS ONE’s publication criteria as it currently stands. Therefore, we invite you to submit a revised version of the manuscript that addresses the points raised during the review process.

The reviewers are supportive and have asked for some minor revisions to strengthen the paper. I trust the authors can move forwards in addressing these remarks.

Please submit your revised manuscript by Aug 21 2026 11:59PM. If you will need more time than this to complete your revisions, please reply to this message or contact the journal office at plosone@plos.org. When you're ready to submit your revision, log on to https://www.editorialmanager.com/pone/ and select the 'Submissions Needing Revision' folder to locate your manuscript file.

Please include the following items when submitting your revised manuscript:

- A letter that responds to each point raised by the academic editor and reviewer(s). You should upload this letter as a separate file labeled 'Response to Reviewers'.
- A marked-up copy of your manuscript that highlights changes made to the original version. You should upload this as a separate file labeled 'Revised Manuscript with Track Changes'.
- An unmarked version of your revised paper without tracked changes. You should upload this as a separate file labeled 'Manuscript'.

If you would like to make changes to your financial disclosure, please include your updated statement in your cover letter. Guidelines for resubmitting your figure files are available below the reviewer comments at the end of this letter.

If applicable, we recommend that you deposit your laboratory protocols in protocols.io to enhance the reproducibility of your results. Protocols.io assigns your protocol its own identifier (DOI) so that it can be cited independently in the future. For instructions see: https://journals.plos.org/plosone/s/submission-guidelines#loc-laboratory-protocols. Additionally, PLOS ONE offers an option for publishing peer-reviewed Lab Protocol articles, which describe protocols hosted on protocols.io. Read more information on sharing protocols at https://plos.org/protocols?utm_medium=editorial-email&utm_source=authorletters&utm_campaign=protocols.

As the corresponding author, your ORCID iD is verified in the submission system and will appear in the published article. PLOS supports the use of ORCID, and we encourage all coauthors to register for an ORCID iD and use it as well. Please encourage your coauthors to verify their ORCID iD within the submission system before final acceptance, as unverified ORCID iDs will not appear in the published article. *Only*  the individual author can complete the verification step; PLOS staff *cannot*  verify ORCID iDs on behalf of authors.

We look forward to receiving your revised manuscript.

Kind regards,

Maria J Berghs, PhD

Academic Editor

PLOS One

Journal Requirements:

When submitting your revision, we need you to address these additional requirements.

1.Please ensure that your manuscript meets PLOS ONE's style requirements, including those for file naming. The PLOS ONE style templates can be found at

https://journals.plos.org/plosone/s/file?id=wjVg/PLOSOne_formatting_sample_main_body.pdf and

https://journals.plos.org/plosone/s/file?id=ba62/PLOSOne_formatting_sample_title_authors_affiliations.pdf

2. Please note that funding information should not appear in any section or other areas of your manuscript. We will only publish funding information present in the Funding Statement section of the online submission form. Please remove any funding-related text from the manuscript.

3. Thank you for stating the following financial disclosure:

“This study was funded through Q Exchange by the Health Foundation and NHS England”

Please state what role the funders took in the study.  If the funders had no role, please state: "The funders had no role in study design, data collection and analysis, decision to publish, or preparation of the manuscript."

If this statement is not correct you must amend it as needed.

Please include this amended Role of Funder statement in your cover letter; we will change the online submission form on your behalf.

4. We note that you have indicated that there are restrictions to data sharing for this study. PLOS only allows data to be available upon request if there are legal or ethical restrictions on sharing data publicly. For more information on unacceptable data access restrictions, please see http://journals.plos.org/plosone/s/data-availability#loc-unacceptable-data-access-restrictions.

Before we proceed with your manuscript, please address the following prompts:

a) If there are ethical or legal restrictions on sharing a de-identified data set, please explain them in detail (e.g., data contain potentially identifying or sensitive patient information, data are owned by a third-party organization, etc.) and who has imposed them (e.g., a Research Ethics Committee or Institutional Review Board, etc.). Please also provide contact information for a data access committee, ethics committee, or other institutional body to which data requests may be sent.

b) If there are no restrictions, please upload the minimal anonymized data set necessary to replicate your study findings to a stable, public repository and provide us with the relevant URLs, DOIs, or accession numbers. For a list of recommended repositories, please see

https://journals.plos.org/plosone/s/recommended-repositories. You also have the option of uploading the data as Supporting Information files, but we would recommend depositing data directly to a data repository if possible.

We will update your Data Availability statement on your behalf to reflect the information you provide.

5. If the reviewer comments include a recommendation to cite specific previously published works, please review and evaluate these publications to determine whether they are relevant and should be cited. There is no requirement to cite these works unless the editor has indicated otherwise.

6. Please review your reference list to ensure that it is complete and correct. If you have cited papers that have been retracted, please include the rationale for doing so in the manuscript text, or remove these references and replace them with relevant current references. Any changes to the reference list should be mentioned in the rebuttal letter that accompanies your revised manuscript. If you need to cite a retracted article, indicate the article’s retracted status in the References list and also include a citation and full reference for the retraction notice.

[Note: HTML markup is below. Please do not edit.]

Reviewers' comments:

Reviewer's Responses to Questions

**Comments to the Author**

1. Is the manuscript technically sound, and do the data support the conclusions?

Reviewer #1: Yes

Reviewer #2: Yes

**********

2. Has the statistical analysis been performed appropriately and rigorously?

Reviewer #1: N/A

Reviewer #2: N/A

**********

3. Have the authors made all data underlying the findings in their manuscript fully available?

The PLOS Data policy

Reviewer #1: Yes

Reviewer #2: No

**********

4. Is the manuscript presented in an intelligible fashion and written in standard English?

Reviewer #1: Yes

Reviewer #2: Yes

**********

Reviewer #1: Many thanks for the opportunity to review this manuscript.

Noting the critically of being able to identify those most at risk of drug related deaths / overdose situations, the authors have developed a ‘trigger checklist’ to estimate the extent of risk in real time and as a practical tool for pro-active assertive outreach in this instance in the ED setting. Given an earlier demonstration of the checklists likely positive impact in community settings, it is a very understandable attempt to elucidate why ED teams showed a relatively low application of the list. There is also the added complexity of the relatively rural setting.

The CMO methodology is appropriate (for ‘setting specific’ evaluations) and well described as are the emergent theories in the results section. The checklists relative simplicity and low threshold (or high sensitivity) are to be commended, though it would be useful to know if the investigators attempted any form of sensitivity analysis (even in the abstract where the likely impact of increasing and deceasing sensitivity might be considered). The extrapolation that 3/4s of those using drugs in the area were not known engaged with services served to further highlight the importance of the project.

Overall, the report presents a very engaging and practical narrative and serves to highlight the sort of actions that are important (relationships unsurprisingly). It is the very context specificity of the benefits (which the authors by no means shy away from) including the variable attitudes to consent which raise the most questions about wider generalisability. Having refined their methods in the course of looking at this setting therefore the authors are well placed to look at generalisability to other settings and other regions (e.g. the larger central belt populations), where the stigma of limited anonymity is less prevalent, but would the relative patient numbers result in a prohibitive work-load in the short term?.

Lastly have the authors considered adapting the tool for prison and justice settings?

Reviewer #2: Thank you for the opportunity to review this realist evaluation of relating to the evaluation of the use of a Trigger Checklist in a rural ED in Scotland. For transparency, I am a qualitative researcher who is familiar with realist methods.

I found the paper clearly written and carefully planned and presented. The aim is clearly stated, and I find that this is well answered by the analysis. I have no ethical concerns.

The ‘problem’ the study is addressing, and the potential transferability and relevance of the work to other contexts, is made clear in the introduction. Moreover, the CMO configurations are presented at a level of detail that is appropriate and useful to allow application to other contexts. The transferability is particularly supported by the use of relational coordination theory.

Realist evaluation is an appropriate methodological choice for the aims of the work. This appears to have been conducted rigorously. The information provided in the Results aligns with the realist analysis process described in the methods. This is appropriately supported by the appendices I’d like to see with a realist review.

I have two small suggestions for improvement:

1. The use of a Project Advisory Group to develop initial programme theories was a strength – was this group consulted to comment on refined/final theories? (Or perhaps the study participants?) If so, please could this be made clear in the paper, and if not, I suggest you discuss this as a limitation. Confirmation from those who understand the lived experience of the intervention is important in realist work and would strengthen the credibility of conclusions.

2. I note the team is (appropriately) a mix of clinical and academic staff. Given the number of subjective decisions necessary within such a review (especially during testing the CMOs), you might consider a short section on team reflexivity to help the reader situate the analysis and aid transparency about how the researchers reduced bias and used reflexive practice in the analytical process.

**********

what does this mean?). If published, this will include your full peer review and any attached files.

If you choose “no”, your identity will remain anonymous but your review may still be made public.

**Do you want your identity to be public for this peer review?**  For information about this choice, including consent withdrawal, please see our Privacy Policy

Reviewer #1: No

Reviewer #2: No

**********

[NOTE: If reviewer comments were submitted as an attachment file, they will be attached to this email and accessible via the submission site. Please log into your account, locate the manuscript record, and check for the action link "View Attachments". If this link does not appear, there are no attachment files.]

To ensure your figures meet our technical requirements, please review our figure guidelines: https://journals.plos.org/plosone/s/figures

You may also use PLOS’s free figure tool, NAAS, to help you prepare publication quality figures: https://journals.plos.org/plosone/s/figures#loc-tools-for-figure-preparation.

NAAS will assess whether your figures meet our technical requirements by comparing each figure against our figure specifications.

# Author comment: Author response to Decision Letter 1

**DOI:** 10.1371/journal.pone.0358880.r002

14 Aug 2026

7th August 2026

Dear Professor Berghs

Manuscript ID PONE-D-25-67347 Implementation of the Trigger Checklist and Assertive Outreach to Reduce Drug-Related Harm in the Emergency Department: A Realist Evaluation

Thank you very much for coordinating the further revision of our manuscript, which we hope will be considered for publication in PLOS ONE.

Please find details of responses to reviewer’s and editorial comments below.

Editorial Comments

1.Please ensure that your manuscript meets PLOS ONE's style requirements, including those for file naming. The PLOS ONE style templates can be found at

https://journals.plos.org/plosone/s/file?id=wjVg/PLOSOne_formatting_sample_main_body.pdf and

https://journals.plos.org/plosone/s/file?id=ba62/PLOSOne_formatting_sample_title_authors_affiliations.pdf

Response

All formatting updated in accordance with the Manuscript Body Formatting Guidelines and Title, Author, Affiliations Formatting Guidelines.

2. Please note that funding information should not appear in any section or other areas of your manuscript. We will only publish funding information present in the Funding Statement section of the online submission form. Please remove any funding-related text from the manuscript.

Response

The Funding Statement has been removed from the manuscript.

3. Thank you for stating the following financial disclosure: “This study was funded through Q Exchange by the Health Foundation and NHS England”

Please state what role the funders took in the study. If the funders had no role, please state: "The funders had no role in study design, data collection and analysis, decision to publish, or preparation of the manuscript." If this statement is not correct you must amend it as needed. Please include this amended Role of Funder statement in your cover letter; we will change the online submission form on your behalf.

Response

This study was funded through Q Exchange by the Health Foundation and NHS England (statement removed from main manuscript). Role of Funder for online submission “The funders had no role in study design, data collection and analysis, decision to publish, or preparation of the manuscript.”

4. We note that you have indicated that there are restrictions to data sharing for this study. PLOS only allows data to be available upon request if there are legal or ethical restrictions on sharing data publicly. For more information on unacceptable data access restrictions, please see http://journals.plos.org/plosone/s/data-availability#loc-unacceptable-data-access-restrictions. Before we proceed with your manuscript, please address the following prompts:

a) If there are ethical or legal restrictions on sharing a de-identified data set, please explain them in detail (e.g., data contain potentially identifying or sensitive patient information, data are owned by a third-party organization, etc.) and who has imposed them (e.g., a Research Ethics Committee or Institutional Review Board, etc.). Please also provide contact information for a data access committee, ethics committee, or other institutional body to which data requests may be sent.

b) If there are no restrictions, please upload the minimal anonymized data set necessary to replicate your study findings to a stable, public repository and provide us with the relevant URLs, DOIs, or accession numbers. For a list of recommended repositories, please see

https://journals.plos.org/plosone/s/recommended-repositories. You also have the option of uploading the data as Supporting Information files, but we would recommend depositing data directly to a data repository if possible.

We will update your Data Availability statement on your behalf to reflect the information you provide.

Response

Although the dataset was anonymised prior to analysis, there remains a potential risk of participant re-identification due to the small size of the rural communities included in the study. Several participants occupy specific professional or community roles that are held by one or a small number of individuals within these communities. As a result, the sharing of even de-identified qualitative data could inadvertently enable identification of participants when combined with contextual information. The study was conducted under ethical approval granted by the University of the Highlands and Islands Ethics Committee (ID: ETH2425-0561), and participants provided informed consent on the understanding that their information would remain confidential. The approved participant information and consent form stated:

“I understand that any information recorded in the interview will remain confidential and no information that identifies me as an individual will be made publicly available.”

Making the dataset publicly available would therefore be inconsistent with the assurances of confidentiality provided to participants and approved by the Ethics Committee. For these ethical reasons, we do not consider public sharing of the dataset to be appropriate.

If you have any further questions or wish to contact an independent person to whom any questions may be directed, please contact:

UHI Research Ethics Officer

University of the Highlands and Islands Research Ethics Committee (REC)

University of the Highlands and Islands, 12b Ness Walk, Inverness, IV3 5SQ.

Telephone: 00 441463-279349

Email: research.ethics@uhi.ac.uk

5. If the reviewer comments include a recommendation to cite specific previously published works, please review and evaluate these publications to determine whether they are relevant and should be cited. There is no requirement to cite these works unless the editor has indicated otherwise. No recommendation to cite specific work in reviewers’ feedback.

6. Please review your reference list to ensure that it is complete and correct. If you have cited papers that have been retracted, please include the rationale for doing so in the manuscript text, or remove these references and replace them with relevant current references. Any changes to the reference list should be mentioned in the rebuttal letter that accompanies your revised manuscript. If you need to cite a retracted article, indicate the article’s retracted status in the References list and also include a citation and full reference for the retraction notice.

Response

References are complete and correct.

To ensure your figures meet our technical requirements, please review our figure guidelines: https://journals.plos.org/plosone/s/figures

You may also use PLOS’s free figure tool, NAAS, to help you prepare publication quality figures: https://journals.plos.org/plosone/s/figures#loc-tools-for-figure-preparation.

NAAS will assess whether your figures meet our technical requirements by comparing each figure against our figure specifications.

Response

The figure has been reformatted to meet the journal requirements using NASS.

Reviewer 1 Comments

The checklists relative simplicity and low threshold (or high sensitivity) are to be commended, though it would be useful to know if the investigators attempted any form of sensitivity analysis (even in the abstract where the likely impact of increasing and deceasing sensitivity might be considered). The extrapolation that 3/4s of those using drugs in the area were not known engaged with services served to further highlight the importance of the project.

Reviewer 1 Responses

We thank the reviewer for this observation. The Trigger Checklist was developed pragmatically through collaboration between experienced Drug and Alcohol practitioners and individuals with lived experience of recovery, with the primary aim of maximising identification of potentially vulnerable individuals who may benefit from outreach. A formal sensitivity analysis was not undertaken as part of this service evaluation/project, and we acknowledge this as a limitation. However, all completed Trigger Checklists were reviewed by a qualified Drug and Alcohol Practitioner prior to outreach activity to assess appropriateness and risk. Practitioner feedback indicated that only a very small number of referrals (fewer than five cases during the project) were subsequently considered unsuitable for outreach. While these observations do not constitute a formal assessment of sensitivity or specificity, they suggest that the checklist functioned as intended in identifying individuals potentially in need of engagement. We have added text to the manuscript acknowledging the absence of formal sensitivity analysis and recommending this as an important area for future evaluation of the checklist (see lines 658-666).

Having refined their methods in the course of looking at this setting therefore the authors are well placed to look at generalisability to other settings and other regions (e.g. the larger central belt populations), where the stigma of limited anonymity is less prevalent, but would the relative patient numbers result in a prohibitive work-load in the short term?

Response

We thank the reviewer for this observation. From a realist perspective, we would not necessarily expect the same context–mechanism–outcome configurations to operate in different settings. Rather, future work would test whether mechanisms identified in this study, such as the value of relational outreach, trust-building, and concerns regarding information sharing, remain important under different contextual conditions. While issues relating to anonymity and stigma may be less pronounced in larger urban populations, alternative contextual influences may become more important. We also agree that feasibility warrants exploration. The continuation of the service in this area was facilitated through organisational reconfiguration and the subsequent creation of two outreach worker posts. Urban areas may face different workforce and capacity challenges due to higher referral volumes, making evaluation of scalability and resource requirements an important next phase of testing and refinement. A quality improvement project is currently underway to test implementation of the Trigger Checklist within an urban Emergency Department setting, which may provide valuable insights into capacity requirements and inform our next realist study.

Reviewer 2 Comments

3. Have the authors made all data underlying the findings in their manuscript fully available?

The PLOS Data policy requires authors to make all data underlying the findings described in their manuscript fully available without restriction, with rare exception (please refer to the Data Availability Statement in the manuscript PDF file). The data should be provided as part of the manuscript or its supporting information, or deposited to a public repository. For example, in addition to summary statistics, the data points behind means, medians and variance measures should be available. If there are restrictions on publicly sharing data—e.g. participant privacy or use of data from a third party—those must be specified.

Response

See response to point 4 of the editorial comments.

1. The use of a Project Advisory Group to develop initial programme theories was a strength – was this group consulted to comment on refined/final theories? (Or perhaps the study participants?) If so, please could this be made clear in the paper, and if not, I suggest you discuss this as a limitation. Confirmation from those who understand the lived experience of the intervention is important in realist work and would strengthen the credibility of conclusions.

Reviewer 2 Responses

We thank the reviewer for this important observation. The Project Advisory Group was involved in the development and refinement of the initial programme theories; however, the refined and final programme theories were not subsequently presented to the group. This was primarily due to time constraints within the project and challenges securing participation from clinical staff given competing service demands. We agree that stakeholder validation of refined programme theories can enhance the credibility of realist findings and provide an opportunity to assess the resonance of interpretations with lived and professional experience. We have therefore acknowledged the absence of final theory refinement with the Project Advisory Group as a limitation and recommend this as an important component of future realist evaluations. See lines 667-675 in the discussion section.

2. I note the team is (appropriately) a mix of clinical and academic staff. Given the number of subjective decisions necessary within such a review (especially during testing the CMOs), you might consider a short section on team reflexivity to help the reader situate the analysis and aid transparency about how the researchers reduced bias and used reflexive practice in the analytical process.

Response

We thank the reviewer for this valuable suggestion. Reflexive discussions were embedded throughout the analysis, although this was not sufficiently described in the manuscript. The multidisciplinary team, comprising academic and clinical members, met weekly during analysis to discuss emerging findings, challenge interpretations, and refine programme theories and CMO configurations. Particular emphasis was placed on retroductive reasoning, with team members debating plausible underlying mechanisms and alternative explanations for observed outcomes. These discussions supported reflexivity, consensus-building, and consideration of different professional perspectives. We have now added a short reflexivity section to the manuscript (see line 269-276 in methods).

We are grateful for the feedback and believe it has improved our paper.

We look forward to hearing from you.

Sincerely,

Dr Michelle Beattie

On behalf of DR HOPE Team

Attachment Submitted filename: DRHOPE_ResponseReviewers_July26.doc

# Peer review: Decision Letter 1

**DOI:** 10.1371/journal.pone.0358880.r003

**Authors:** Maria Berghs

8 Sep 2026

Implementation of the Trigger Checklist and Assertive Outreach to Reduce Drug-Related Harm in the Emergency Department: A Realist Evaluation

PONE-D-25-67347R1

Dear Dr. Beattie,

We’re pleased to inform you that your manuscript has been judged scientifically suitable for publication and will be formally accepted for publication once it meets all outstanding technical requirements.

Within one week, you’ll receive an e-mail detailing the required amendments. When these have been addressed, you’ll receive a formal acceptance letter and your manuscript will be scheduled for publication.

An invoice will be generated when your article is formally accepted. Please note, if your institution has a publishing partnership with PLOS and your article meets the relevant criteria, all or part of your publication costs will be covered. Please make sure your user information is up-to-date by logging into Editorial Manager at Editorial Manager® and clicking the ‘Update My Information' link at the top of the page. For questions related to billing, please contact billing support.

If your institution or institutions have a press office, please notify them about your upcoming paper to help maximize its impact. If they’ll be preparing press materials, please inform our press team as soon as possible -- no later than 48 hours after receiving the formal acceptance. Your manuscript will remain under strict press embargo until 2 pm Eastern Time on the date of publication. For more information, please contact onepress@plos.org.

Kind regards,

Maria J Berghs, PhD

Academic Editor

PLOS One

Reviewers' comments:

Reviewer's Responses to Questions

**Comments to the Author**

Reviewer #2: All comments have been addressed

**********

2. Is the manuscript technically sound, and do the data support the conclusions?

Reviewer #2: (No Response)

**********

3. Has the statistical analysis been performed appropriately and rigorously?

Reviewer #2: (No Response)

**********

4. Have the authors made all data underlying the findings in their manuscript fully available?

The PLOS Data policy

Reviewer #2: (No Response)

**********

5. Is the manuscript presented in an intelligible fashion and written in standard English?

Reviewer #2: (No Response)

**********

Reviewer #2: The authors have effectively addressed the feedback I raised in my earlier review and have made relevant changes to the paper.

**********

what does this mean?). If published, this will include your full peer review and any attached files.

If you choose “no”, your identity will remain anonymous but your review may still be made public.

**Do you want your identity to be public for this peer review?**  For information about this choice, including consent withdrawal, please see our Privacy Policy

Reviewer #2: No

**********

# Editor report: Acceptance letter

**DOI:** 10.1371/journal.pone.0358880.r004

**Authors:** Maria Berghs

PONE-D-25-67347R1

PLOS One

Dear Dr. Beattie,

I'm pleased to inform you that your manuscript has been deemed suitable for publication in PLOS One. Congratulations! Your manuscript is now being handed over to our production team.

At this stage, our production department will prepare your paper for publication. This includes ensuring the following:

* All references, tables, and figures are properly cited

* All relevant supporting information is included in the manuscript submission,

* There are no issues that prevent the paper from being properly typeset

You will receive further instructions from the production team, including instructions on how to review your proof when it is ready. Please keep in mind that we are working through a large volume of accepted articles, so please give us a few days to review your paper and let you know the next and final steps.

Lastly, if your institution or institutions have a press office, please let them know about your upcoming paper now to help maximize its impact. If they'll be preparing press materials, please inform our press team within the next 48 hours. Your manuscript will remain under strict press embargo until 2 pm Eastern Time on the date of publication. For more information, please contact onepress@plos.org.

You will receive an invoice from PLOS for your publication fee after your manuscript has reached the completed accept phase. If you receive an email requesting payment before acceptance or for any other service, this may be a phishing scheme. Learn how to identify phishing emails and protect your accounts at https://explore.plos.org/phishing.

If we can help with anything else, please email us at customercare@plos.org.

Thank you for submitting your work to PLOS One and supporting open access.

Kind regards,

PLOS One Editorial Office Staff

on behalf of

Dr. Maria J Berghs

Academic Editor

PLOS One
